# The promise of anti-angiogenic cancer therapy

**DOI:** 10.1054/bjoc.1999.0991

**Published:** 2000-02

**Authors:** M K Oehler, R Bicknell

**Affiliations:** Molecular Angiogenesis Laboratory, Imperial Cancer Research Fund, Institute of Molecular Medicine, University of Oxford, John Radcliffe Hospital, Oxford, OX3 9DS, UK


*British Journal of Cancer* (2000) **82**(4), 749–752
© 2000 Cancer Research Campaign
DOI: 10.1054/ bjoc.1999.0991, available online at http://www.idealibrary.com on IDEAL®

Commentary

### The promise of anti-angiogenic cancer therapy

**MK Oehler and R Bicknell**

Molecular Angiogenesis Laboratory, Imperial Cancer Research Fund, Institute of Molecular Medicine, University of Oxford, John Radcliffe Hospital, Oxford OX3 9DS, UK

It is now over 200 years since the British surgeon John Hunter first used the term angiogenesis to describe the growth of new blood vessels in the developing reindeer antler (Hunter, 1787) and some 30 years since the possibility of antagonizing angiogenesis as a novel anticancer strategy first became recognized by the scientific community. It is only in the last 5 years, however, that the field of (anti)angiogenesis research has undergone an explosive growth in activity (Figure 1). A primary reason for this has been an increasing optimism amongst researchers that anti-angiogenesis does indeed, at the present time, represent one of the most exciting opportunities for the development of completely new approaches to the treatment of cancer. The purpose of this commentary is to give an assessment of where we stand at present and of the future potential for angiogenic inhibitors in cancer therapy.

### CURRENT STATUS OF LEADING ANGIOGENESIS INHIBITORS

At least 30 angiogenesis inhibitors are currently being assessed in clinical trials. Most are in clinical phase I or II studies. A few, however, have progressed to phase III evaluation, potentially leading to Federal Drug Administration (FDA) approval (Table 1).

Leading anti-angiogenic targets that have been identified are (1) the inhibition of matrix metalloproteinases, (2) antagonism of the VEGF pathway of angiogenic induction, and (3) inhibition of the $\alpha_v\beta_3$-integrin–vitronectin interaction that is pivotal in mediating endothelial cell adhesion to the extracellular matrix during neovascularization.

Some of the most advanced angiogenesis inhibitors currently being evaluated in clinical trials are matrix metalloproteinase (MMP) inhibitors. Marimastat (British Biotech, Annapolis, MD, USA) was the first MMP inhibitor to be involved in rigorous clinical trials. In a phase III study incorporating 400 patients with advanced pancreatic cancer, marimastat showed no single therapy benefit over gemcitabine, the ‘drug of choice’. Nevertheless, marimastat at 25 mg twice a day was as effective as gemcitabine and appeared to have an improved therapeutic index at lower doses (5 mg or 10 mg) with fewer side-effects. It is clear that further studies of marimastat will be needed before a complete assessment of the efficacy, tolerability and dose regimen can be made. To this end, nine randomized controlled studies of marimastat in a range of solid tumours (pancreatic, non-small-cell lung, breast cancers) are ongoing and the current expectation is that these data will be available within the next 3 years.

*Received 30 July 1999*
*Accepted 10 September 1999*

*Correspondence to*: R Bicknell

Other metalloproteinase inhibitors in advanced trials are Bay 12-9566 (Bayer, West Haven, CT, USA) and Ag3340 (Agouron, La Jolla, CA, USA). Several international phase III clinical trials using Bay 12-9566 against solid tumours including lung, ovarian and pancreatic cancer are being conducted. In addition, the National Cancer Institute (NCI) is currently performing phase I studies designed to evaluate a possible use of Bay 12-9566 in combination regimens with doxorubicin, fluorouracil or low-dose leucovorin. There are several phase III clinical trials underway to evaluate AG3340 alone or in combination with the anticancer drugs paclitaxel/carboplatin for the treatment of non-small-cell lung cancer and mitoxantrone/prednisone for hormone-refractory prostate cancer. It has been shown that the anti-tumour efficacy of AG3340 is associated with maintenance of a minimum plasma concentration but not total daily dose, exposure or peak plasma concentrations (Brekken et al, 1999).

Attempts to abrogate the angiogenic activity of vascular endothelial growth factor (VEGF) have varied from inactivation of VEGF itself by using, for example, antibodies (Mordenti et al, 1999) or soluble receptors to specific inhibition of the VEGF receptor tyrosine kinase (Lin et al, 1998). The latter includes ZD4190, an anilino quinazoline derivative that specifically inhibits the VEGF receptor 2 (KDR) tyrosine kinase and has shown widespread anti-tumour activity in in vivo animal models following oral administration (Hennequin et al, 1999; Ogilvie et al, 1999). Another VEGF receptor tyrosine kinase inhibitor showing much promise is SU5416 (Sugen Inc., CA, USA).

The interaction of the angiogenic endothelial cell with extracellular vitronectin mediated via the $\alpha_v$ integrin is crucial during angiogenesis. It follows that antibodies to the $\alpha_v\beta_3$ integrin are strongly anti-angiogenic and mouse monoclonals have been humanized as ‘Vitaxin’ to permit clinical trials. Preliminary results have shown stable disease or shrinkage in eight of 14 late-stage cancer patients. No side-effects have been observed so far (Eliceiri and Cheresh, 1999).

### ANGIOSTATIN AND ENDOSTATIN, EMERGING ANGIOGENESIS INHIBITORS

An area of intense current interest is that of potent naturally occurring inhibitors of angiogenesis being encrypted within larger molecules with no angiogenic activity but having other functions. Proteolytic cleavage releases the active material. Such molecules include angiostatin, a fragment of plasminogen (O’Reilly et al, 1997); endostatin, a fragment of collagen type 18 (O’Reilly et al, 1997); a 16 kDa fragment of prolactin; and a fragment of thrombospondin. Attention has been most focused on endostatin, which is able to bring about successive waves of substantial tumour

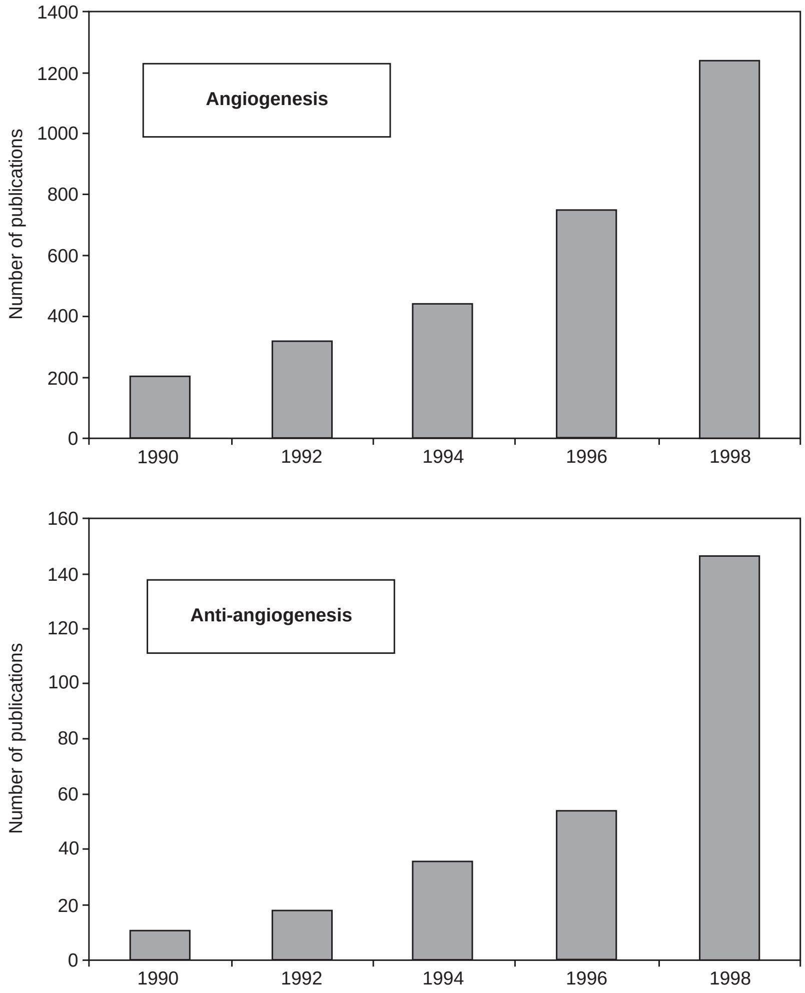

**Figure 1** Publications in angiogenesis and anti-angiogenesis between 1990 and 1998

regression in animal models without the appearance of drug resistance (Boehm et al, 1997).

One year ago, a front page article in the *New York Times* (Kolata, 1998) initiated speculation that cancer could be treated with angiostatin and endostatin. That article, and coverage by other media, generated intense public interest in angiogenesis inhibitors and a subsequent controversial and emotional debate (Wadman, 1998; Cohen, 1999; Harris, 1999; Rowe, 1999). The controversy was fueled as studies on endostatin in laboratories outside Boston were unable to confirm the endostatin results, showing only a slight growth retardation of Lewis lung carcinoma (a difficult tumour to treat with chemotherapeutic agents) in mice. Members of the NCI went to Folkman's laboratory in Boston to clarify why the results differed from those in the original study. In Boston, subsequent results were consistent with previous experiments, showing striking inhibition of tumour growth. Variations in experimental techniques between the two laboratories, such as injecting mice, as well as storage, handling and purification of endostatin, are assumed to have been responsible for the previously observed lack of agreement in results.

The NCI's success with mouse endostatin – just a few months after it had been announced publicity that it could not replicate the results – allowed the Institute to initiate plans for the testing of human endostatin in patients, pending full-scale toxicology studies. In early fall of 1999 two sites were expected to conduct phase I studies with approximately 15–25 patients each in patients with solid tumours, including lung, breast, colon and prostate carcinoma. To launch clinical studies it has been necessary to scale up the production of endostatin. By applying a yeast expression system it is now possible to produce soluble human endostatin at quantities sufficient for clinical assessment in man (Sim et al, 1999).

Animal studies with angiostatin and endostatin have so far been only with transplanted tumours, which show a different biology when compared to organ-specific, spontaneous tumours and are not necessarily accurate predictors of what will happen in natural human cancers. Thus, a transgenic mouse model of pancreatic islet carcinogenesis (RIP1-Tag2) was used as a model to examine the effect of several angiogenesis inhibitors on multistage tumorigenesis (Bergers et al, 1999). Apart from endostatin, angiostatin and a combination of both, AGM-1470 (TNP470; a fumagillin derivative which is thought to inhibit endothelial cell proliferation by irreversible binding to the enzyme methionylaminopeptidase-2) (Sin et al, 1997) and BB-94 (batimastat; a matrix metalloproteinases inhibitor) (Talbot and Brown, 1996) were tested for their

© 2000 Cancer Research Campaign

**Table 1** Most advanced anti-angiogenic agents in clinical trials (source: NCI Cancer Trials – http://cancertrials.nci.nih.gov)

| Drug | Trial | Mechanism |
|---|---|---|
| Marimastat | Phase III against pancreas, non-small-cell lung, breast cancers | Synthetic MMP inhibitor |
| Bay 12-9566 | Phase III against lung, ovary and pancreatic cancers | Synthetic MMP inhibitor |
| AG3340 | Phase III against non-small-cell lung; phase III against prostate cancer | Synthetic MMP inhibitor |
| Thalidomide | Phase II against Kaposi's sarcoma, glioblastoma, breast, prostate and lung cancers | Unknown |
| Anti-VEGF antibody | Phase II/III against lung, breast, prostate, colorectal and renal cancers | Monoclonal antibody to VEGF |
| SU5416 | Phase I/II against Kaposi's sarcoma, phase I/II against metastatic colorectal cancer, and phase I/II against advanced malignancies | Blocks VEGF receptor signalling |
| CAI | Phase II/III against ovarian, non-small-cell lung, and renal cell cancers | Inhibitor of calcium influx |

MMP, matrix metalloproteinase; VEGF, vascular endothelial growth factor.

anti-angiogenic potency in a prevention, intervention and regression trial in the RIP-Tag mice. The four angiogenesis inhibitors examined showed distinct efficacy profiles varying from about 60% to 85% depending on the stage of carcinogenesis being targeted. However, none of the agents tested completely prevented the angiogenic switch, blocked the growth of small tumours, or fully resolved lethal tumour burden. These results suggest that the prevention and treatment of human spontaneous organ-specific malignancies with anti-angiogenic agents is going to be more complex and difficult than was originally anticipated. For example, it is not possible to predict if human tumours are going to respond in vivo to human endostatin as do mouse tumours to mouse endostatin.

The story of angiostatin and endostatin shows that much has yet to be learned about anti-angiogenic agents. Several years after the discovery of angiostatin and endostatin, recent reports began to give insight into the mechanism of anti-angiogenic action of those agents. Angiostatin has been shown to inhibit matrix-enhanced plasminogen activation, resulting in reduced invasive activity (Stack et al, 1999). Angiostatin's antiproliferative effect was reported to be mediated by binding to the $\alpha/\beta$-subunits of ATP synthase (Moser et al, 1999). The last observation is noteworthy as it might be possible to develop small molecules that could mimic angiostatins effect on the ATP synthase-binding protein. Smaller molecules would reduce the problem of immunogenicity, might be more easy to synthesize and might be taken orally. Less is known about the mechanism of action of endostatin but it is believed to induce apoptosis of endothelial cells by reducing anti-apoptotic proteins like Bcl-2 (Dhanabal et al, 1999*b*).

### OTHER ANGIOGENESIS INHIBITORS

It may not be the agents currently in trials that work best in the end. Several hundred angiogenesis inhibitors have been identified and the list is mushrooming, including exotic substances like those extracted from green tea (Cao and Cao, 1999). It is possible that some of the newly identified agents are going to be more potent than the currently known drugs. Although the chance of an anti-angiogenic agent moving into standard medical practice is estimated to be in the order of 1:10 000, a 'gold-rush' atmosphere has developed to detect or develop such a compound as the potential market is comparable to antibiotics and chemotherapeutics, that is of the order of billions of dollars per year (Brem, 1998).

Some previously known drugs have also been shown to be angiogenesis inhibitors. For example thalidomide, a drug with a tarnished past, achieved a comeback after its antiangiogenic properties were identified. (D'Amato, 1994), and it is almost no surprise that aspirin with its pleiotropic effects has been identified as an angiogenesis inhibitor (Tsujii et al., 1998).

### ANTI-ANGIOGENESIS AND CONVENTIONAL ANTICANCER THERAPIES

Many traditional cancer therapies probably have an anti-angiogenic component. Thus, chemotherapeutic agents such as the taxanes and camptothecins have anti-angiogenic properties that may, at least in part, account for their efficacy as anti-tumour agents (Belotti et al, 1996). It is possible that low-dose standard chemotherapeutic regimens may inhibit angiogenesis without being cytotoxic to the tumour. Anti-oestrogen therapy for the prevention or adjuvant treatment of breast cancer may also be mediated by affecting vascularity as tamoxifen has been shown to inhibit angiogenesis (Van der Schaft et al, 1999).

Further evidence for the anti-angiogenic activity of conventional as well as experimental cancer therapies comes from a growing number of studies that have shown that damage of blood vessels precede or accompanies tumour regression after radiation therapy, hyperthermia, photodynamic therapy or administration of a variety of biological response modifiers such as interferon, tumour necrosis factor, interleukins or endotoxin (Baillie, 1995). Finally, it is also known that many oncogenes modulate the expression of angiogenic factors, such as VEGF, and thus therapies targeting these genes may also be effective through the inhibition of angiogenesis (Rak et al, 1995).

### DESIGNING CLINICAL TRIALS OF ANGIOGENESIS INHIBITORS

There are important differences between anti-angiogenic clinical trials and traditional trials of cytotoxics (Kerbel and Pluda, 1999). In phase I trials most of the anti-angiogenic agents have been exceptionally well tolerated, lacking many of the side-effects associated with conventional cancer chemotherapies (neutropenia, nausea and vomiting etc.). Due to this lack of measurable toxicity, it has been difficult to define the maximum tolerated dose and to identify a recommended drug dose. Further, in phase II studies it has proven a challenge to assess the effectiveness of angiogenic drugs. These relate to the observations that most of those agents do not necessarily cause tumour shrinkage but induce tumour dormancy leading to stable disease. Exceptions are agents used for vascular targeting (Huang et al, 1997) and the more recently described natural inhibitors, such as angiostatin and endostatin. Measuring time-to-progression is a parameter to determine stable disease but it tends to be a heterogeneous time point, with great

© 2000 Cancer Research Campaign interpersonal variation (Kerbel and Pluda, 1999). Two methods have, however, been usefully applied to assess responsiveness to anti-angiogenic therapy: (i) measurement of serum levels of angiogenic peptides and (ii) magnetic resonance imaging to detect contrast uptake and washout in tumours. Due to the difficulties of phase II trials, it seems likely that many anti-angiogenesis drugs will proceed rapidly from phase I to phase III.

### CONCLUSIONS

For many years the perceived role for angiogenesis inhibitors in the clinic was either in the adjuvant situation or in combination with conventional cytotoxic's permitting use of lower doses of the latter. Recently, however, the arrival of new angiogenesis inhibitors such as endostatin that achieve substantial tumour regression points to a potentially greater role for angiogenesis inhibitors in oncology. Clinical application of anti-angiogenic agents looks an increasingly realistic prospect. Clearly, the next few years will see a period of intense research into the clinical potential for inhibitors of angiogenesis in the treatment of cancer.

### REFERENCES

Baillie CT (1995) Tumour vasculature – a potential therapeutic target. *Anti Cancer Drugs* **6**: 438

Belotti D, Vergani V, Drudis T, Borsotti P, Pitelli MR, Viale G, Giavazzi R and Taraboletti G (1996) The microtubule-affecting drug paclitaxel has antiangiogenic activity. *Clin Cancer Res* **2**: 1843–1849

Bergers G, Javaherian K, Lo KM, Folkman J and Hanahan D (1999) Effects of angiogenesis inhibitors on multistage carcinogenesis in mice. *Science* **284**: 808–812

Boehm T, Folkman J, Browder T and O'Reilly MS (1997) Antiangiogenic therapy of experimental cancer does not induce acquired drug resistance. *Nature* **390**: 404–407

Brekken J, Zou H, Kolis S, Wood A, Webber S, Appelt K and Shalinsy DR (1999) Antitumor efficacy of AG3340 associated with maintenance of minimum effective plasma concentrations and not total daily dose, exposure or peak plasma concentrations. *Proc Am Ass Cancer Res* **40**: 441 (abstract)

Brem S (1998) Angiogenesis antagonists: current clinical trials. *Angiogenesis* **2**: 9–20

Cao Y and Cao R (1999) Angiogenesis inhibited by drinking tea. *Nature* **398**: 381

Cohen J (1999) Behind the headlines of endostatin's ups and downs. *Science* **283**: 1250–1251

D'Amato RJ (1994) Thalidomide is an inhibitor of angiogenesis. *Proc Natl Acad Sci USA* **91**: 4082

Dhanabal M, Ramchandran R, Volk R, Stillman IE, Lombardo M, Iruela-Arispe ML, Simons M and Sukhatme VP (1999*a*) Endostatin: yeast production, mutants, and antitumor effect in renal cell carcinoma. *Cancer Res* **59**: 189–197

Dhanabal M, Ramchandran R, Waterman MJ, Lu H, Knebelmann B, Segal M and Sukhatme VP (1999*b*) Endostatin induces endothelial cell apoptosis. *J Biol Chem* **274**: 11721–11726

Eliceiri BP and Cheresh DA (1999) The role of alphav integrins during angiogenesis: insights into potential mechanisms of action and clinical development. *J Clin Invest* **103**: 1227–1230

Harris RF (1999) Gristle for the mill. *Curr Biol* **9**: R232

Hennequin LF, Thomas AP, Johnstone C, Ple P, Stokes ESE, Ogilvie DJ, Dukes M and Wedge SR (1999) ZD4190: The design and synthesis of a novel, orally active VEGF receptor tyrosine kinase inhibitor. *Proc Am Ass Cancer Res* **40**: 457 (abstract)

Huang XM, Molema G, King S, Watkins L, Edgington TS and Thorpe PE (1997) Tumor infarction in mice by antibody-directed targeting of tissue factor to tumor vasculature. *Science* **275**: 547–550

Hunter J (1787) *Lectures on the Principles of Surgery*, Vol. 1.: London

Kerbel RS and Pluda J (1999) Preclinical and clinical aspects of anti-angiogenic strategies to treat cancer. *American Society of Clinical Oncology, 35th Annual Meeting, Atlanta*

Kolata G (1998) A cautious awe greets drugs that eradicate tumors in mice. *The New York Times*, May 3

Lin P, Sankar S, Shan S, Dewhirst MW, Polverini PJ, Quinn TQ and Peters KG (1998) Inhibition of tumor growth by targeting tumor endothelium using a soluble vascular endothelial growth factor receptor. *Cell Growth Differ* **9**: 49–58

Mordenti J, Thomsen K, Licko V, Chen H, Meng YG and Ferrara N (1999) Efficacy and concentration-response of murine anti-VEGF monoclonal antibody in tumor-bearing mice and extrapolation to humans. *Toxicol Pathol* **27**: 14–21

Moser TL, Stack MS, Asplin I, Enghild JJ, Hojrup P, Everitt L, Hubchak S, Schnaper HW and Pizzo SV (1999) Angiostatin binds ATP synthase on the surface of human endothelial cells. *Proc Natl Acad Sci USA* **96**: 2811–2816

Ogilvie DJ, Wedge SR, Dukes M, Kendrew J, Curwen JO, Thomas AP, Hennequin LF, Ple P, Stokes ESE, Johnstone C, Wadsworth P, Richmond GHP and Curry B (1999) ZD4190: An orally administered inhibitor of VEGF signalling with pan-xenograft anti-tumor activity. *Proc Am Ass Cancer Res* **40**: 458 (abstract)

O'Reilly MS (1997) Angiostatin: an endogenous inhibitor of angiogenesis and of tumor growth. *Exs* **79**: 273–294

O'Reilly MS, Boehm T, Shing Y, Fukai N, Vasios G, Lane WS, Flynn E, Birkhead JR, Olsen BR and Folkman J (1997) Endostatin: an endogenous inhibitor of angiogenesis and tumor growth. *Cell* **88**: 277–285

Rak J, Filmus J, Finkenzeller G, Grugel S, Marme D and Kerbel RS (1995) Oncogenes as inducers of tumor angiogenesis. *Cancer Metastasis Rev* **14**: 263–277

Rowe PM (1999) What is all the hullabaloo about endostatin? *Lancet* **353**: 732

Sim BKL, Fogler WE, Zhou XH, Liang H, Madsen JW, O'Reilly MS, Panigrahy D and Fortier AH (1999) Potent inhibition of experimental metastases and primary tumors by recombinant human endostatin that is suitable for human use. *Proc Am Ass Cancer Res* **40**: 4092 (abstract)

Sin N, Meng L, Wang MQ, Wen JJ, Bornmann WG and Crews CM (1997) The anti-angiogenic agent fumagillin covalently binds and inhibits the methionine aminopeptidase, MetAP-2. *Proc Natl Acad Sci USA* **94**: 6099–6103

Stack MS, Gately S, Bafetti LM, Enghild JJ, Soff GA, Moser TL, Asplin I, Hojrup P, Everitt L, Hubchak S, Schnaper HW and Pizzo SV (1999) Angiostatin inhibits endothelial and melanoma cellular invasion by blocking matrix-enhanced plasminogen activation: angiostatin binds ATP synthase on the surface of human endothelial cells. *Biochem J* **340**: 77–84

Talbot DC and Brown PD (1996) Experimental and clinical studies on the use of matrix metalloproteinase inhibitors for the treatment of cancer. *Eur J Cancer* **32a**: 2528–2533

Tsujii M, Kawano S, Tsuji S, Sawaoka H, Hori M and DuBois RN (1998) Cyclooxygenase regulates angiogenesis induced by colon cancer cells. *Cell* **93**: 705–716

Van der Schaft DWJ, Barendsz-Janson AF, Hillen HFP and Griffioen AW (1999) Tamoxifen inhibits human angiogenesis. *Proc Am Ass Cancer Res* **40**: 3004 (abstract)

Wadman M (1998) Cancer 'cure' article stirs up hot debate. *Nature* **393**: 104–105

*© 2000 Cancer Research Campaign*

